# Brain-derived neurotrophic factor serum levels in genetically isolated populations: gender-specific association with anxiety disorder subtypes but not with anxiety levels or Val66Met polymorphism

**DOI:** 10.7717/peerj.1252

**Published:** 2015-10-29

**Authors:** Davide Carlino, Ruggiero Francavilla, Gabriele Baj, Karolina Kulak, Pio d’Adamo, Sheila Ulivi, Stefania Cappellani, Paolo Gasparini, Enrico Tongiorgi

**Affiliations:** 1 Psychiatric Clinic, Department of Surgical and Medical Sciences, University of Trieste, Trieste, Italy; 2 Department of Life Sciences, University of Trieste, Trieste, Italy; 3 Department of Surgical and Medical Sciences, University of Trieste, Trieste, Italy; 4 Institute for Maternal and Child Health, IRCCS “Burlo Garofolo”, Trieste, Italy

**Keywords:** Neurotrophins, Brain-derived neurotrophic factor, Serum biomarkers, Val66Met polymorphism, Genome wide analysis, Anxiety, Sex factors, Generalized Anxiety Disorder, Specific Phobia, Social Phobia

## Abstract

Anxiety disorders (ADs) are disabling chronic disorders with exaggerated behavioral response to threats. This study was aimed at testing the hypothesis that ADs may be associated with reduced neurotrophic activity, particularly of Brain-derived neurotrophic factor (BDNF), and determining possible effects of genetics on serum BDNF concentrations. In 672 adult subjects from six isolated villages in North-Eastern Italy with high inbreeding, we determined serum BDNF levels and identified subjects with different ADs subtypes such as Social and Specific Phobias (PHSOC, PHSP), Generalized Anxiety Disorder (GAD), and Panic Disorder (PAD). Analysis of the population as a whole or individual village showed no significant correlation between serum BDNF levels and Val66Met polymorphism and no association with anxiety levels. Stratification of subjects highlighted a significant decrease in serum BDNF in females with GAD and males with PHSP. This study indicates low heritability and absence of any impact of the Val66Met polymorphism on circulating concentrations of BDNF. Our results show that BDNF is not a general biomarker of anxiety but serum BDNF levels correlate in a gender-specific manner with ADs subtypes.

## Introduction

Anxiety disorders (ADs) are disabling medical disorders affecting 6% of men and 13% of women in the USA and 21% of the general population in Italy and France (US Census Bureau, 2005; Alonso et al., 2004; Kessler et al., 2005a; Kessler et al., 2005b; Leray et al., 2011). ADs frequently occur early in life, have a chronic course and adversely affect the prognosis of other medical illnesses (Kessler et al., 2005a; Van Noorden et al., 2012). ADs comprise a collection of syndromes characterized by exaggerated fear responses to perceived threats. Such threats extend to a wide range of situations in Generalized Anxiety Disorder (GAD), and to specific ones, such as social evaluation in Social Phobia (PHSOC). ADs commonly occur along with other mental or physical illnesses, including mood disorders and alcohol or substance abuse, which may mask anxiety symptoms or make them worse (Yu et al., 2013).

A current hypothesis is that ADs, in analogy with mood disorders, may be associated with altered neurotrophic activity (Chen et al., 2006; Duman & Monteggia, 2006; Hashimoto, 2010; Tsai, 2003). Accumulating evidence suggests the neurotrophin Brain Derived Neurotrophic Factor (BDNF) is reduced in mood disorders (Duman & Monteggia, 2006; Tsai, 2003). Increased BDNF expression in the forebrain is at the base of the antidepressant effect of many drugs and physical exercise acting through neurotrophic enhancement of neuronal stem cells survival and stimulation of network connectivity (Baj et al., 2012). In transgenic mice, overexpression of BDNF has a facilitating effect on anxiety-like behaviour possibly due to increased spine density in amygdale (Govindarajan et al., 2006). In another study in which anxiety-related personality traits were correlated with BDNF polymorphism Val66Met, higher levels of trait anxiety were found in subjects bearing the Val/Val BDNF allele compared to Val/Met and Met/Met genotypes having reduced BDNF availability and secretion at synapses (Chen et al., 2006).

Interestingly, BDNF can be found not only in brain but also in peripheral tissues, including blood. The opportunity to detect BDNF in serum has important clinical implications because it provides the possibility to evaluate brain functions avoiding invasive methods (Carlino et al., 2011; Elfving, Plougmann & Wegener, 2010; Trajkovska et al., 2007). Accordingly, several studies focused on BDNF levels in blood as potential biomarker for major depression disorder (MDD) and reported lower BDNF blood level in both serum and plasma of drug-free patients compared to healthy controls (Bocchio-Chiavetto et al., 2010; Karege et al., 2005; Lee & Kim, 2008; Sen, Duman & Sanacora, 2008; Shimizu et al., 2003). In contrast, few studies were conducted to explore serum BDNF as a biomarker in patients with anxiety disorders. In particular, reduced serum levels of BDNF have been found in patients with panic disorders but no alterations were found for other ADs (Kobayashi et al., 2005; Strohle et al., 2010). A study reported a weak association between reduced serum BDNF and anxiety, but only in females (Molendijk et al., 2011). The aim of the present research was to explore whether BDNF may represent a biomarker of ADs by comparing serum BDNF levels among adult patients with different subtypes of ADs which were Generalized Anxiety Disorder (GAD), Specific Phobia (PHSP), Panic Attack Disorder (PAD), Social Phobia (PHSOC) and healthy donors, in a large population sample (*n* = 672 subjects). Moreover, we estimated the heritability (the proportion of variance due to genetic factors) of the blood levels of BDNF and performed an association analysis to check for the association between rs6265 (responsible for the Val66Met polymorphism) and BDNF levels. For both analysis we used already available whole genome genotyping data on the adult population of the Genetic Park (GP) from the Region “Friuli-Venezia Giulia” consisting of 6 isolated villages in North–Eastern Italy, characterized by reduced genetic variability (Esko et al., 2013).

## Materials & Methods

### Study population

The present study sample (672 subjects) was drawn from a large population of adults investigated in the study “Genetic Park of Friuli Venezia Giulia (GP—Friuli Venezia Giulia). This project was launched in Spring 2009 and involved, on a voluntary basis, inhabitants of the “six geographical isolates” of Friuli Venezia Giulia, (i.e., Sauris, Erto/Casso, San Martino, Illegio, Clauzetto, Resia). The aim of GP was to analyze the environmental and genetic factors implicated in the pathogenesis of several diseases such as mood disorders, deafness, osteoporosis and diabetes. The geographic isolates met the criteria defining “genetic isolates” as separate geographical location with high endogamy rate, language barrier, few surnames, few founders, low emigration and immigration rates. The study was approved by the Ethics Committee of the IRCCS-Burlo Garofolo of Trieste. Participants were informed about the study and those who provided a written consent were included, according to the recommendations of the declaration of Helsinki and the Italian DL no 675 of the 31-12-1996. All participants received a structured diagnostic interview using the Composite International Diagnostic Interview (CIDI) in order to assess current and lifetime DSM-IV-TR diagnoses. A total of 252 subjects fulfilled DSM-IV-TR criteria for the diagnosis of ADs. Exclusion criteria were: diagnosis of substance abuse, neurodegenerative disease, major depression, schizophrenia or bipolar depression.

### BDNF assessment in GP—Friuli Venezia Giulia

Blood was collected from all 672 participants between 7:30 and 11:30 AM and immediately transferred to laboratory sites for serum preparation or DNA extraction. Upon collection, blood samples were split in two aliquots, one in tubes with anti-coagulant for DNA analysis and the other in normal tubes for clotting and isolation of the serum samples. During transfer from the villages to the laboratory site, blood samples for DNA extraction were conserved at −30 °C, while samples for serum collection were at +4 °C. After centrifugation at 2.000 g for 10 min, serum samples were stored at −80 °C and BDNF measurement was performed in duplicate using the Emax Immuno assay System (Promega) according to manufacturer and expressed in ng/mL. Samples were not acidified before testing. The reaction was stopped 10–15 min later with 1M HCl solution, and the absorbance was immediately measured at 450 nm (Promega Glomax Multi Detection System). BDNF concentrations were determined from the regression line for the BDNF standard ranging from 7.8 to 500 pg/ml BDNF.

### Psychiatric assessment

The presence and severity of symptoms of anxiety were assessed using a composite psychometric battery consisting of: (1) the Structure Clinical interview for DSM-IV-TR (SCID–I); (2) the 14-item Hamilton Anxiety Rating Scale (HAM-A). Battery tests were administered by a neuropsychiatrist qualified and experienced in administering these interviews (D.C.). Each interview was lasting approximately 60 min. Twenty percent of diagnostic interviews were reviewed by a second neuropsychiatrist to confirm reliability of diagnostic categories.

### Genotyping

DNA was extracted from all 672 blood samples according to the method described in Esko et al., 2013. All DNA samples were genotyped with ILLUMINA arrays (ILLUMINA HumanCNV370-Quad Beadchip) using standard protocols. After genotyping, all the samples with a call rate <0.95, MAF <0.05 and markers with call-rate <0.95 or not in Hardy–Weinberg equilibrium were removed.

### Statistical analysis

All data were analyzed with R v.3.1.0. Variables’ distribution was tested for normality. Differences in BDNF serum concentrations between the villages were determined by one/two-way analysis of variance (ANOVA). Linear regression analysis was performed to explore the relationship between serum BDNF levels and HAM-A scores in GAD, PHSP, PAD, PHSOC. Mann–Whitney *U* test 1 tail was used to explore whether there were different serum BDNF levels among control and patient groups in all villages. We chose the 1 tail Mann–Whitney U test because, according to previous studies, only decreased BDNF levels are expected to be linked to mood disorders and pathological types of anxiety. The heritability of BDNF levels was estimated using the restricted maximum likelihood analysis implemented in GCTA (Yang et al., 2011) and association analysis corrected for kinship was performed with GenABEL v.1.8. (Aulchenko et al., 2007).

## Results

### Serum levels of BDNF, socio-demographics and anxiety

**Figure 1:**
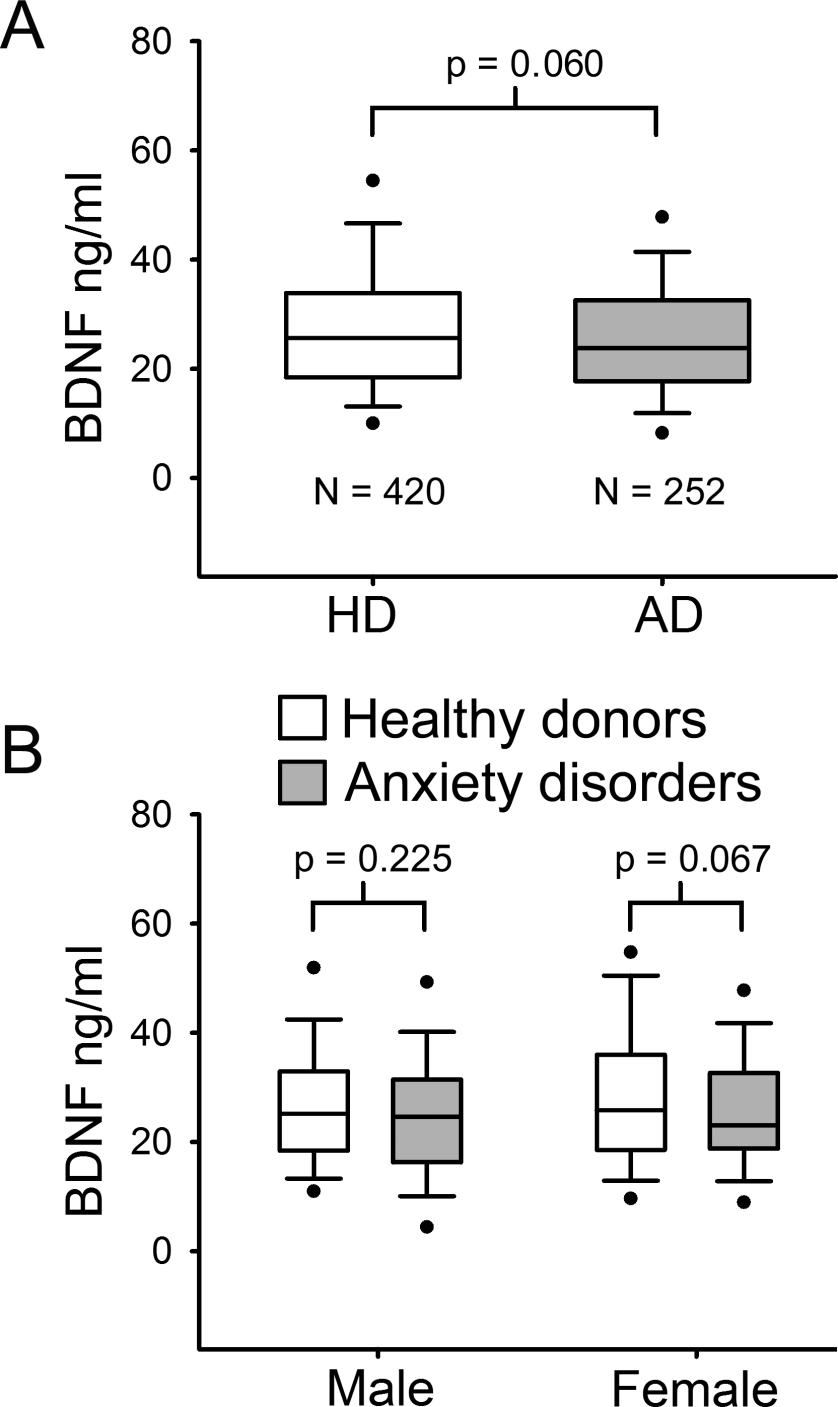
Serum BDNF level in Healthy donors (*n* = 420) and Anxiety disorders Patients (*n* = 252). (A) BDNF values are not normally distributed. Mann–Whitney *U* Test 1-tail, revealed no significant statistical difference between the two groups (*p* = 0.060). (B) Serum BDNF level in male and female groups; *n*, number of subjects [healthy donors, patients]; male [219, 71], female [201, 181].

The population (*n* = 672) was stratified in healthy donors (HD) and patients with ADs. Table 1 describes baseline socio-demographic characteristics of this sample. HD had a median age of 47 years ranging from 18 to 76 years, whereas patients with ADs had a median age of 48 years within a minimum of 19 to a maximum of 73 years. As serum BDNF levels were not normally distributed, Mann–Whitney U test was used to compare median values among the different groups. No significant difference in serum BDNF levels was found between HD and AD groups (*p* = 0.06 MW-U Test; Fig. 1A). Indeed, in the HD group the median BDNF value was 27.58 ng/ml, ranging from 3.33 ng/ml to 66.85 ng/ml, and the AD group showed a quite similar median BDNF value (25.62 ng/ml, ranging from 3.10 ng/ml to 63.78 ng/ml) (Table 1). To determine whether there was any significant difference related to gender, BDNF levels were compared in males and females in both HD and AD groups (Fig. 1B). Mann–Whitney U test comparison between HD and AD males showed no significant difference in BDNF levels (*p* = 0.225); and no significant difference was found in female subjects between the two study groups (*p* = 0.067).

**Table 1:** Baseline socio-demographic characteristics of the study sample.

	Healthy donors	AD	*p*-value
*N* of subjects per group/total sample	420/672	252/672	–
Male/female	219/201	71/181	–
Age median value	47	48	0.548
Range	18–76	19–73	
Serum BDNF median ng/ml	27.58	25.62	Total subject
25%–75% percentile	18.46–33.85	17.79–32.54	0.060
			Male/female
Range of detection	3.33–66.85	3.10–63.78	0.225/0.067

**Notes.**

AD anxiety disorders SD standard deviation

### Heritability and association analysis

**Figure 2:**
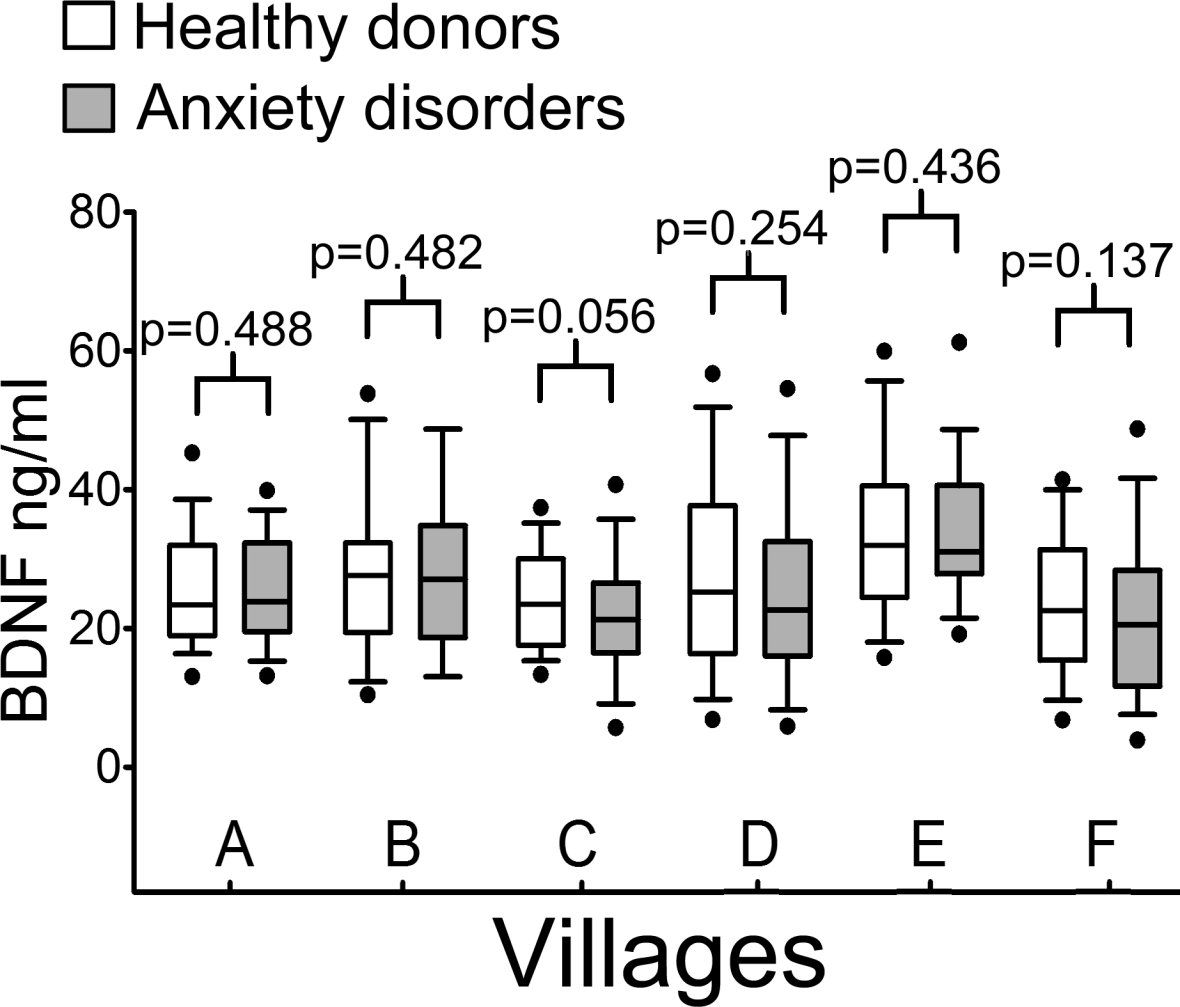
Serum BDNF levels in six villages of the Genetic Park of Friuli Venezia Giulia; *n*, number of subjects [healthy controls, patients]. (A) Clauzetto [69, 54], (B) Erto [27, 18], (C) Illegio [75, 50], (D) Resia [139, 49], (E) San Martino [73, 38], (F) Sauris [40, 43]. A comparison Mann–Whitney *U* Test 1-tail shows no difference among the control and patient groups in all villages.

As previously reported by Esko et al., 2013, the population sample analyzed showed a high degree of inbreeding and/or founder effects. The presence of high inbreeding may result in a different incidence of specific disorders with respect to the outbred population and may highlight the presence of single genetic polymorphisms with high impact on serum BDNF levels. To investigate the effect of heritability on ADs incidence and serum BDNF levels, we carried out an analysis of serum BDNF levels in the six villages of GP, considering the gender. The analysis of the six isolated villages showed no significant difference among the median value of healthy donors and ADs groups (Mann–Whitney U test 1-tail; Clauzetto *p* = 0.488, Erto *p* = 0.482, Illegio *p* = 0.056, Resia *p* = 0.254, San Martino *p* = 0.436, Sauris *p* = 0.137) (Fig. 2).

In order to check for a possible contribution of genetics to the basal BDNF levels in the serum, we use the software GCTA. The result is that only 20% of the trait depends from genetic factors (V(G)/Vp = 0.203262). We also tried to calculate the contribution of every single chromosome to the trait in order to check for a major locus, but the results are not reliable due to the small sample size. After heritability estimation, we used genotypes data to perform an association analysis between BDNF levels and rs6265 polymorphism controlling for relationship between samples and using an additive model. The availability of whole genome genotyping data allowed us to conduct an association analysis corrected for genomic kinship, avoiding any stratification bias potentially correlated with the use of related samples. Association analysis using age, sex and village as covariate, was not able to find an association with rs6265, the most studied polymorphism of BDNF (*p*-value of 0.26). In addition, the possible effect of the SNP rs6265 (Val66Met) was investigated in relation to BDNF levels and presence of anxiety disorders. Regression analysis with village reveal that this SNP has no effect on blood level of BDNF or that the effect is too small to be detected.

### Serum levels of BDNF in anxiety disorders subtypes

**Figure 3:**
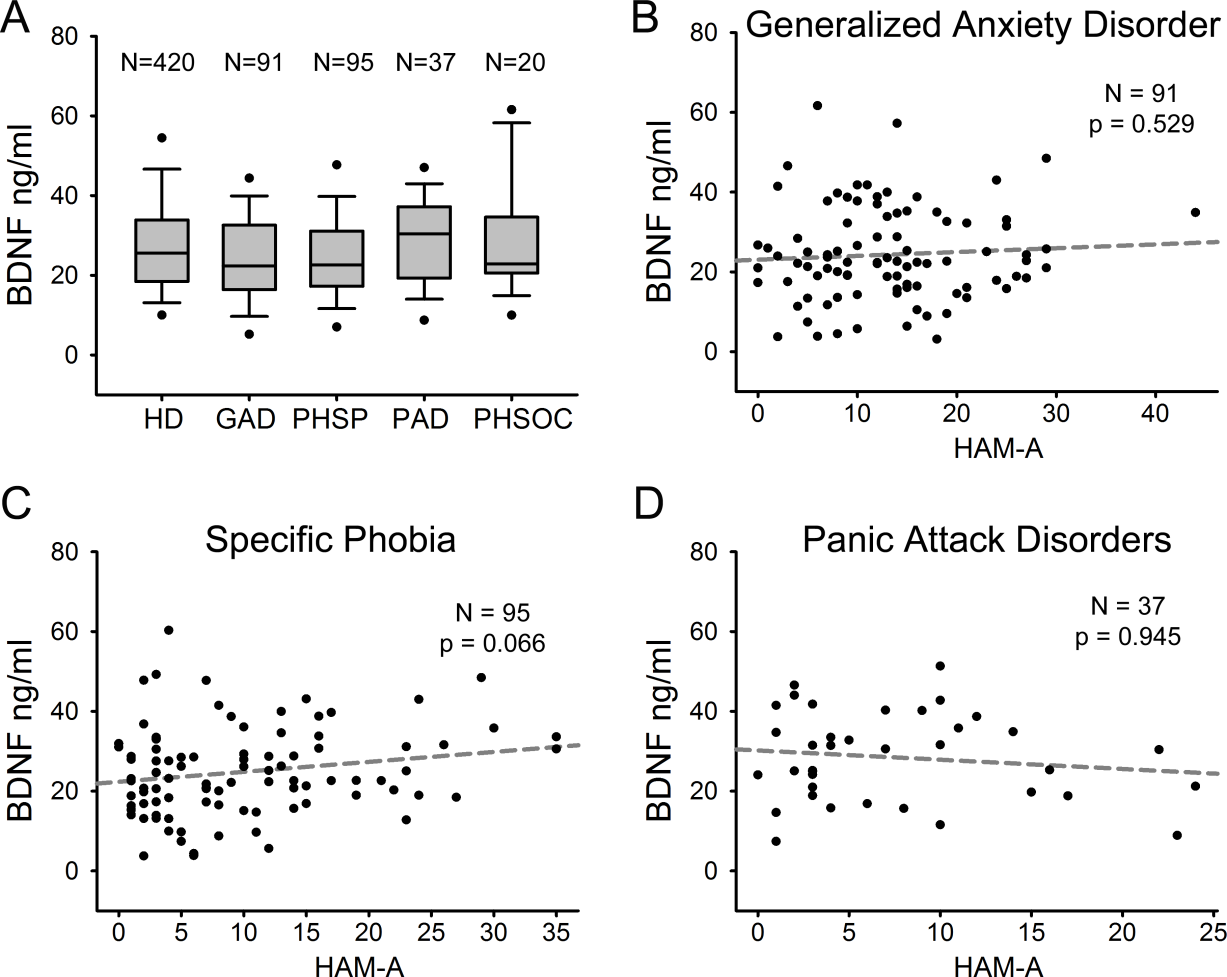
Serum BDNF in different diagnostic categories of Anxiety Disorder. (A) Box Plot Graph showing the distribution of serum BDNF levels between different diagnostic categories of anxiety disorders. (B–D) Linear regression analysis of serum BDNF levels and severity of disease in 3 different categories of anxiety disorders measured with HAM-A (Hamilton Anxiety Rating Scale).

We compared serum BDNF levels among patients with different ADs. Figure 3A shows serum BDNF levels distribution in HD and the different AD diagnostic categories including GAD (*n* = 91), PHSP (*n* = 95), PAD (*n* = 37), and PHSOC (*n* = 20). No significant difference was found in serum BDNF levels for GAD Vs HD (*p* = 0.051); PHSP Vs HD (*p* = 0.114); PAD Vs HD (*p* = 0.376); or PHSOC Vs HD (*p* = 0.913; Mann–Whitney *U* test 2 tail). OCD was not considered because of the low number of patients affected in the population investigated. In all forms of ADs, serum BDNF levels resulted independent from the severity of disease (GAD *p* = 0.529; PHSP *p* = 0.066; PAD *p* = 0.945, linear regression analysis Figs. 3B–3D).

**Figure 4:**
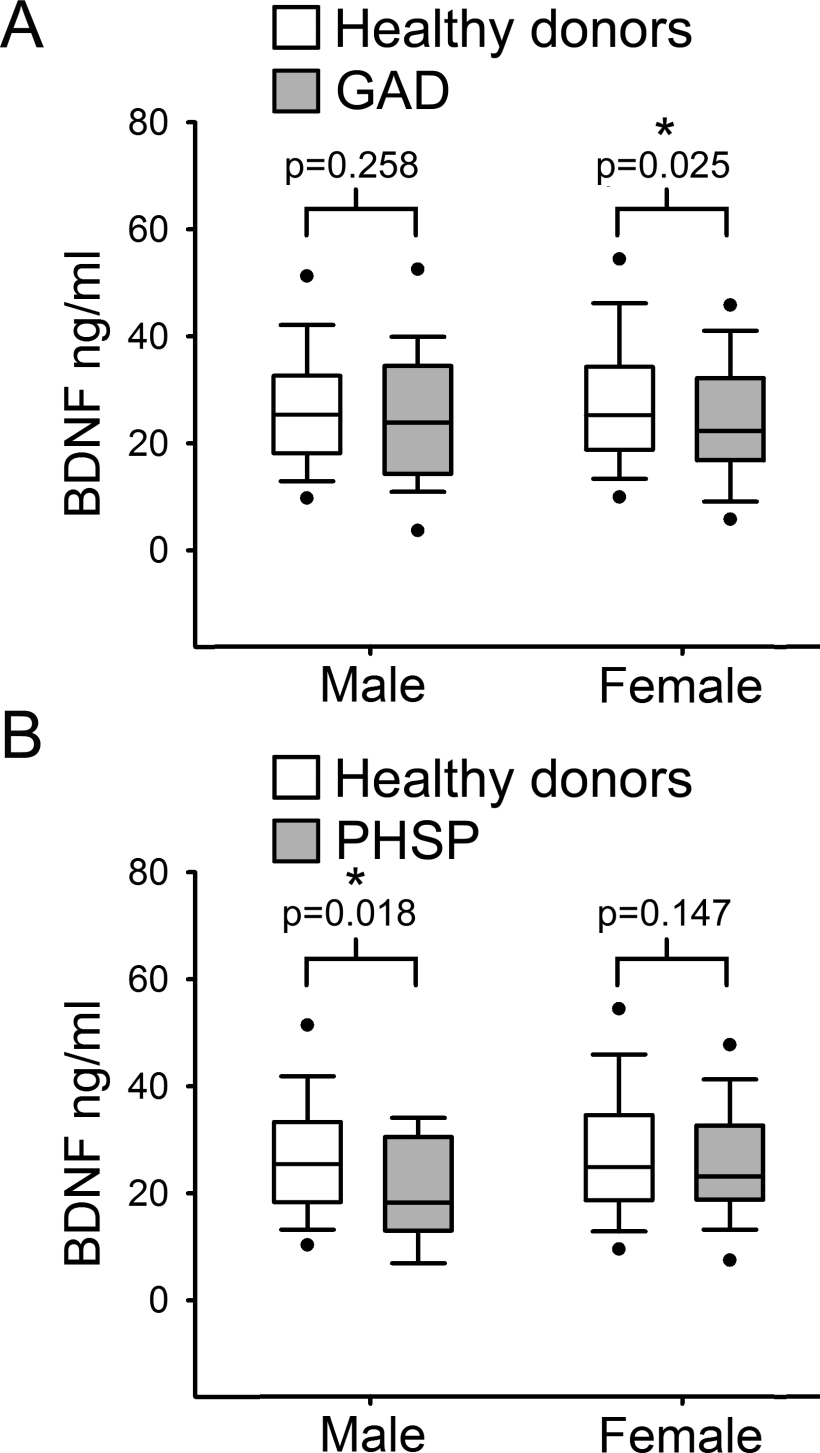
Analysis of BDNF levels gender differences in Generalized Anxiety Disorders and Specific Phobia. (A) Serum BDNF level in male and female groups affected by Generalized Anxiety Disorder; *n*, number of subjects [healthy donors, patients]; male [219, 28], female [201, 63]. Comparison using Mann–Whitney *U* Test 1-tail shows significant statistical difference in serum BDNF level between the two groups in female subjects (^∗^, *p* = 0.025). (B) Serum BDNF level in male and female groups affected by Specific Phobia; male [219, 15], female [201, 82]. Comparison Mann–Whitney *U* Test 1-tail shows significant statistical difference in serum BDNF level between the two groups in male subjects (^∗^, *p* = 0.018). Abbreviation: HD, Healthy Donors; GAD, Generalized Anxiety Disorders; PHSP, Specific Phobia.

Finally, serum BDNF levels were compared separately in males and females from the healthy donor group versus each ADs category. Comparison showed a statistically significant reduction of serum BDNF level in GAD female subjects with respect to female HD (Fig. 4A; *p* = 0.025, Mann–Whitney *U* test 1 tail) but not in males. In contrast, in males affected by PHSP there was a significant reduction in serum BDNF levels in comparison to healthy donors, but not in females (Fig. 4B; male [219, 15] *p* = 0.018, female [201, 63]; Mann–Whitney *U* test 1 tail). No significant gender difference was found for patients with PAD and PHSOC.

## Discussion

In this study, we tested the hypothesis that serum BDNF levels could be a potential biomarker for the different subtypes of ADs. Analysis of the population as a whole or single village excluded any correlation between serum BDNF levels and ADs as a whole or as single diagnostic category. No correlation between BDNF levels and severity of anxiety symptoms was found. However, considering the high level of hidden substructure of the populations, when patients were stratified for both gender and disorder subtype we found a significant decrease in serum levels of BDNF in GAD females. Genome wide analysis of the population as a whole or individual village excluded significant correlations between serum BDNF levels and the Val66Met polymorphism.

Measurement of circulating BDNF is considered of great interest as a possible biomarker of psychiatric and neurodegenerative diseases. However, the origin of circulating BDNF is currently a matter of discussion because in humans and in rodents, BDNF is produced by various body tissues such as smooth muscle cells of blood vessels, skeletal muscles, kidney, liver and brain (Aid et al., 2007; Pruunsild et al., 2007). In blood, BDNF is produced by lymphocytes but not by megakaryocites, however, it is taken up by platelets that function as storage and release system (Karege et al., 2005). The current focus of the debate is whether circulating BDNF levels reflect or not the brain levels of this neurotrophin. Radiolabeled BDNF injected in the jugular vein or in the brain ventricle readily crosses the blood–brain barrier in both directions (Pan et al., 1998) In addition, it has been shown that physical exercise induces an increase of serum BDNF levels which is contributed by 70% from the brain (Rasmussen et al., 2009). Thus, measurement of serum levels of BDNF may provide information on brain diseases and blood samples may be drawn from living subjects to monitor disease progression or treatment efficacy. It has been proposed that socio-demographics variables such as age may affect quantification of BDNF in serum. However, controversial data have been reported on the relationship between serum BDNF and age. Ziegenhorn et al., 2007 reported a weak age-associated decrease in serum BDNF in a group of elderly above 70 years of age while a recent large population study reported that BDNF levels decline with age in women, whereas in men remain stable (Bus et al., 2012). Other studies found no correlation between age and BDNF in serum or whole blood (Bus et al., 2011; Lommatzsch et al., 2005; Trajkovska et al., 2007). Another confirmation of these findings comes from animal studies showing that brain BDNF concentration did not change with age (Webster et al., 2002). In the sample analyzed in this study there was no effect of age on serum BDNF levels.

A major outcome of this study is that we found no genetic effects or on serum levels of BDNF and frequency of ADs, as evidenced by a little heritability of serum BDNF levels and no significant association of the Val66Met polymorphism in genetically isolated, highly inbred populations. These populations were chosen because high inbreeding can increase the frequency of rare variants with high impact on the phenotype and, thanks to the linkage disequilibrium, these variants can be detected analyzing common variants in the same haploblock with standard arrays. Complex traits are characterized by important environmental and polygenic contributions. Isolated populations which are characterized by a reduced variability in both these factors, can facilitate the identification of both the genetic and environmental component because: (a) there is a reduction for the confounder effect of the environment; (b) the relatedness reduces the genetic differences between case and controls improving a lot the power to detect a true association. For this reason isolated populations are often used to detect polymorphisms and environmental factors that contribute to complex traits also in the general population, but that are easiest to detect in this context.

A heritability estimate for BDNF serum level was already reported by Terracciano et al. (2013) who conducted a genome-wide analysis based on data from a larger samples (ca. 1,600 subjects) and derived an estimate of ca 50%. The same paper also conducted a meta-analysis of thirteen studies (for a total of ca. 4,700 samples) that already investigated the association between Val66Met and serum BDNF level, similarly resulting in no significant association with rs6265. In a further study on a genetically isolated population, the same authors also conducted a SNP-by-SNP GWAS finding a number of suggestive signals, although all below Bonferroni threshold, with some of them pointing for a role of TrkC in regulation BDNF expression (Terracciano et al., 2013).

Every GWA study must face the problem of multiple testing due to the enormous number of independent test required. For this reason the *p*-value required for an association at genome level is <5^∗^10^−8^. For complex traits in which every genetic variant gives a modest contribution to the phenotype, this level of significance can be achieved only analyzing a large number of samples (as in Terracciano et al., 2013). Our sample size is relatively small for a successful GWA analysis, so we preferred to test the validity of an association with a specific genetic variant (i.e., the rs6265 SNP) rather than test all the 370 k variants in the array. However, we used all the genomic data only to perform the correction for the relatedness between samples (using the genomic kinship) and heritability. Both these parameters require whole genome data to be correctly calculated. Our data indicate a 20% heritability which, due to the relatively small population sample of this study did not allow us to identify any significant locus which can affect serum BDNF levels. In a previous study, on a large sample of 6157 subjects, the estimated heritability for serum CRP level is between 25–40%, and 18 genome-wide significant loci were identified with evidence of replication for eight of them (Dehghan et al., 2011). The poor heritability indicates that there isn’t a major locus with high impact on BDNF levels. It is likely that BDNF levels is a multifactorial trait with several loci involved, each of them with modest impact on the phenotype.

Our results are in agreement with a recent study by Molendijk and colleagues (2011) who reported no overall differences for serum BDNF levels between healthy subjects and patients with ADs. Other studies reported lower BDNF levels in PAD patients compared to HDs (Strohle et al., 2010), or no differences, although the PAD patients who were poor responders to a cognitive behavioural therapy showed lower BDNF serum levels (Kobayashi et al., 2005). Taken together, our results and those of previous studies support the idea that reduced BDNF levels cannot represent *per se* a pathophysiological cause of ADs. The absence of significant differences in serum BDNF levels between anxious subjects and healthy donors indicate that BDNF is not a general biomarker of anxiety. On the other hand in rodents, overexpression of the TrkB high affinity receptor for BDNF, leads to increased anxiety indicating that BDNF signalling is associated with anxiety (Koponen et al., 2004). However, since TrkB is an integral membrane receptor, its levels are not measurable in serum.

In this study besides BDNF, we did not investigated the other members of the neurotrophin family which also includes Nerve Growth Factor (NGF), Neurotrophin-3 (NT-3) and Neurotrophin-4/5 (NT-4/5) because the available data indicate that they are not useful as biomarkers of anxiety. Indeed, although it was previously shown that NGF levels raise in the serum of soldiers in proximity to their first parachute jumps, in patients affected by generalized anxiety disorder (GAD), NGF levels were not different from controls (Aloe et al., 1994; Jockers-Scherubl et al., 2007). Similarly, in the serum of depressed adolescents or in the brain of maternally separated mice, two conditions including high anxiety levels, NT-3 levels are not different from controls while no information is available yet on NT-4/5 (Pallavi et al., 2013; Yee et al., 2007).

Concerning gender differences, our findings are in agreement with studies which found no gender difference regarding BDNF serum level (Lang, Hellweg & Gallinat, 2004; Ziegenhorn et al., 2007). However, other studies reported that BDNF in whole blood or plasma was higher in women compared to men (Lommatzsch et al., 2005; Trajkovska et al., 2007). On the other hand, our findings strongly support a specific contribution of low serum levels of BDNF to vulnerability to GAD in females and PHSP in males, which is partially in agreement proposed hypothesis that serum BDNF is altered only in females with multiple types of ADs (Molendijk et al., 2011). Although epidemiologic studies have clearly shown that women are more likely than men to develop anxiety-related disorders, the current evidence does not support that female sex can represent a vulnerability factor of anxiety, per se. Indeed, it has been recently proposed that a second factor of vulnerability should be taken into account: i.e., behavioural inhibition (Catuzzi & Beck, 2014). Behavioral inhibition (BI) is characterized by rapid acquisition of anxiogenic stimuli-induced responses that are resistant to extinction and may be present in both sexes. In this “two hit” model of anxiety vulnerability, females possessing a BI temperament with resistant associations between a given stimulus and a behavioural response, will more likely to develop an anxiety disorder (Catuzzi & Beck, 2014). Besides these considerations, the reason for the gender-specific association of lowered BDNF levels with GAD in females and PHSP in males is presently unknown and warrants future investigations.

## Supplemental Information

10.7717/peerj.1252/supp-1 Supplemental Information 1 Raw data from genetic park The table represents the dataset used to perform the analysis of correlation between BDNF amount quantified in human sera. The data are structured as single line for single sample with the genetic park code, the village of origin, the BDNF quantification, the gender and the different anamnestic assessments (all other columns).
